# The gap between medical faculty’s perceptions and use of e-learning resources

**DOI:** 10.1080/10872981.2017.1338504

**Published:** 2017-06-16

**Authors:** Kyong-Jee Kim, Youngjoon Kang, Giwoon Kim

**Affiliations:** ^a^ Department of Medical Education, Dongguk University School of Medicine, Goyang, Republic of Korea; ^b^ Department of Medical Education, Jeju National University School of Medicine, Jeju, Republic of Korea; ^c^ Department of Emergency Medicine, Soonchunhyang University School of Medicine, Bucheon, Republic of Korea

**Keywords:** Medical education, e-learning, teaching and learning, faculty perceptions

## Abstract

**Background**: e-Learning resources have become increasingly popular in medical education; however, there has been scant research on faculty perceptions and use of these resources.

**Objective**: To investigate medical faculty’s use of e-learning resources and to draw on practical implications for fostering their use of such resources.

**Design**: Approximately 500 full-time faculty members in 35 medical schools across the nation in South Korea were invited to participate in a 30-item questionnaire on their perceptions and use of e-learning resources in medical education. The questionnaires were distributed in both online and paper formats. Descriptive analysis and reliability analysis were conducted of the data.

**Results**: Eighty faculty members from 28 medical schools returned the questionnaires. Twenty-two percent of respondents were female and 78% were male, and their rank, disciplines, and years of teaching experience all varied. Participants had positive perceptions of e-learning resources in terms of usefulness for student learning and usability; still, only 39% of them incorporated those resources in their teaching. The most frequently selected reasons for not using e-learning resources in their teaching were ‘lack of resources relevant to my lectures,’ ‘lack of time to use them during lectures,’ and ‘was not aware of their availability.’

**Conclusions**: Our study indicates a gap between medical faculty’s positive perceptions of e-learning resources and their low use of such resources. Our findings highlight the needs for further study of individual and institutional barriers to faculty adoption of e-learning resources to bridge this gap.

## Introduction

e-Learning has increasingly garnered attention and has become integral to the educator’s toolbox in medical education [1], especially as technology becomes more pervasive in the lives of students [2]. Accordingly, there are many e-learning resources available for medical students through recent developments of educational technologies and the internet [3,4]. In line with such trends there have been nationwide efforts in Korea to support e-learning in medical education among medical schools, to offer medical students equal access to quality online resources [5]. These resources include clinical cases, images, videos, and online modules and they are developed and peer-reviewed by medical school faculty from 35 medical schools participating in the Korean consortium for e-learning in medical education. These resources are available for free for members of the participating medical schools through an e-learning portal, www.mededu.or.kr [5].

As e-learning resources have now become widely available in medical education, the question of its effectiveness and its utilization are surfacing. Even though e-learning has gained a firm place in medical education, a study by Back et al. [6] indicates that its distribution and promotion is inhomogeneous across medical schools. There have been several studies on medical students’ positive perceptions and extensive use of e-learning resources [3,7–9], but research on faculty perceptions and use of these resources has been limited. Therefore, research on this topic is necessary as faculty perceptions and experiences are fundamental to their adoption of pedagogical change [10]. Herein, we investigated medical faculty’s perceptions and use of e-learning resources for medical education.

## Methods

The sample for our study was from approximately 500 full-time faculty members in 35 medical schools, who were registered users of the e-learning portal set up by the consortium of Korean medical schools described earlier. We invited them to fill out self-administered questionnaires on their perceptions and use of e-learning resources. This 30-item questionnaire included seven items on participant demographics and eight items regarding their perceptions of e-learning, adapted from ‘Learning Object Evaluation Scale for Teachers’ [11]. This survey instrument was developed to evaluate faculty perceptions of e-learning resources and comprised three sub-scales: learning, usability, and engagement. The original instrument was translated into Korean by the authors. Additionally, participant’s overall satisfaction with e-learning resources was measured in two items. Participants rated their responses using a five-point Likert scale (1 = ‘strongly disagree’, 5 = ‘strongly agree’). In another item, participants’ use of e-learning resources was measured by asking the frequency of their use of such resources in their teaching using a five-point Likert scale (1 = ‘never’, 5 = ‘frequently’). Participants were also asked on how they were using these resources and the reasons for not using them, by choosing one or more answers from a list of response options. Additionally, an open-ended item was included to elicit their opinions on barriers to their use of e-learning resources.

The questionnaires were distributed in both online and paper formats during the spring months of 2016. To avoid non-response bias, we made attempts to enhance the response rate by offering financial incentives, sending frequent reminders, and using diverse formats – paper-based and online-based surveys – as suggested by Phillips et al. [12].

Descriptive analysis was performed with the data collected from this study, and reliability analysis was conducted on the sub-scales in the questionnaire to evaluate internal consistency of the items. IBM-SPSS Statistics for Windows, Version 23.0 was used for data analysis.

Our study fell under the general exemption from our institutional review board for educational outcomes data; therefore board approval was not requested. Participation was voluntary, and consent was implied with the return of the survey, as responses were collected anonymously.

## Results

Eighty faculty members from 28 medical schools returned the questionnaires (response rate approximately 16%). Seventy were completed online and 10 were completed using the paper version, which was returned either by postal mail or e-mail. Twenty-two percent of the respondents were female and 78% were male; their ages ranged from 35 to 65 years (M = 45.97 SD = 7.00). The respondents varied in their rank and backgrounds – 34% were assistant professors, 26% were associate professors, and 40% were professors; and they were from 21 different departments in basic and clinical medicine. The majority of participants (69%) had teaching experience of between five and 15 years; 18% had less than five years teaching experience, and 13% had over 15 years experience. The participants’ average years of experience of using e-learning in their teaching were 3.94 (SD = 2.98).

Table 1 presents participant responses on their perceptions of e-learning and the reliability of each sub-scale. The respondents somewhat agreed with the statements that e-learning was useful for student learning, and that it was usable; however, they were neutral on the statement that students engaged in e-learning. Still, participants agreed with the statements that they were satisfied with e-learning.

**Table 1.** Faculty perceptions of e-learning resources (n = 80).

Sub-scales	Mean * (standard deviation)	Reliability (Cronbach’s α)
Usefulness for learning	3.86 (.52)	.85
Usability	3.69 (.55)	.71
Student engagement	3.12 (.71)	.87
Overall satisfaction	4.01 (.72)	.78

*1 = ‘strongly disagree,’ 5 = ‘strongly agree’

In terms of participants’ use of e-learning resources, 38.7% (*n* = 50) reported that they had used these resources either occasionally or frequently. For those who had used e-learning resources, some cited these sources as references in their teaching materials (*n* = 43, 54%), some referred to them when they prepare for their lectures (*n* = 32, 40%), and others used them as pre-class assignments for the students (*n* = 25, 31%). Table 2 presents participants responses on reasons for not using e-learning resources. The most frequently selected responses were ‘lack of resources relevant to my lectures,’ ‘lack of time to use it during lectures,’ and ‘was not aware of its availability.’

**Table 2.** Participant responses on reasons for not using e-learning resources in their teaching (n = 80).

Options	Responses * n (%)
Lack of resources relevant to my lectures	24 (30%)
Lack of time to use it during lectures	22 (27%)
Was not aware of its availability	21 (26%)
No need for non-traditional resources	9 (11%)
Inconvenient to use	9 (11%)
Dissatisfied with the quality of resources	5 (0.6%)
Others	0 (0%)

*multiple responses

In response to the open-ended question, several participants commented on the need for more promotion of the e-learning portal to enhance awareness for both faculty and students, and on how to use its resources, so that they use them more frequently and actively for teaching and learning. Additionally, some called for better organization and development of a more comprehensive repository of learning resources that encompass all of the content areas that pertain to the national standards for the educational objectives for basic medical education.

## Conclusions

Our study indicates the positive perceptions of e-learning resources by medical faculty members in terms of usefulness for student learning and usability. However, our survey respondents were neutral regarding their perceptions of student engagement with e-learning resources. It is speculated that this is due, in part, to faculty members’ lack of understanding of how students are making use of e-learning resources, as they are usually used outside of classroom such as during self-study hours. This was indicated by the participant responses to an open-ended question in the questionnaire.

We found a gap between medical faculty’s perceptions of e-learning resources and their actual use of such resources in their teaching. Medical faculty members were not actively using e-learning resources in their teaching despite their positive perceptions of these resources. Such a gap in medical faculty’s conceptions and practice on e-learning may reflect the challenges in using technology for the delivery of the curriculum in medical education, as suggested by Sandars [13]. Our study indicates that there are three major barriers preventing medical faculty members from adopting and fully incorporating e-learning into their teaching: (1) lack of e-learning resources for their specific content areas, (2) lack of awareness of such resources available for them, and (3) lack of understanding of how to integrate e-learning resources into their teaching. This finding is in line with the literature from higher education settings indicating various factors affecting faculty adoption of technology and new pedagogies in higher education in online or blended learning settings [10].

Limitations to this study need to be acknowledged. This was a preliminary study with a small sample size and a low response rate; the generalizability of this study is potentially limited. As mentioned, we made several efforts to improve the response rate. Moreover, we made efforts to maintain the representativeness of the sample by drawing from a nationwide sample and included respondents who were diverse in their demographics and backgrounds. Although there is no information available on the demographics of medical school faculty in South Korea, it is speculated that the respondents’ demographics in this study is representative of the study sample in terms of gender. Approximately 24% of doctors in the nation are female, and this ratio may be comparable to that of female faculty members in medical schools nationwide, which is similar to that of respondents in this study.

Our study illustrates the gap between faculty perceptions and use of e-learning resources and highlights the needs for institutional support and strategies to bridge this gap. Our findings indicate that various interventions are necessary to raise faculty’s awareness of e-learning resources and to offer faculty development programs on how to effectively incorporate them into their curriculum in ways to make their teaching more student-centered.
